# Structural Health Monitoring of Underground Structures in Reclamation Area Using Fiber Bragg Grating Sensors

**DOI:** 10.3390/s19132849

**Published:** 2019-06-27

**Authors:** Zhen Liu, Pengzhen Liu, Cuiying Zhou, Yuncong Huang, Lihai Zhang

**Affiliations:** 1 School of Civil Engineering, Sun Yat-sen University, No. 135 XinGangXi Road, Guangzhou 510275, China; liuzh8@mail.sysu.edu.cn (Z.L.); liupzh6@mail2.sysu.edu.cn (P.L.); ueit@mail.sysu.edu.cn (Y.H.); 2 Guangdong Engineering Research Centre for Major Infrastructure Safety, Guangzhou 510275, China; 3 Research Center for Geotechnical Engineering and Information Technology, Sun Yat-sen University, No. 135 XinGangXiLu, Guangzhou 510275, China; 4 Department of Infrastructure Engineering, The University of Melbourne, Melbourne, VIC 3010, Australia; lihzhang@unimelb.edu.au

**Keywords:** reclamation area, underground structures, traffic induced vibration, rainfall, tide levels, temperature, crack width, fiber Bragg grating sensors

## Abstract

The long-term structural performance of underground structures in reclamation areas is very sensitive to the vibrations caused by vehicles passing above the structures and environmental factors (e.g., tide levels, rainfall and temperature). In the present study, an integrated remote real-time structural health monitoring system using fiber Bragg grating sensors was developed to assess the structural performance of underground structures. Using a composite road box-type structure project in a reclamation area in Southern China as a case study, the developed real-time system was implemented to investigate the effects of changes in tide levels, rainfall, temperature and vehicle induced vibrations on crack propagation in the structure. The results show that the change in tide levels has little influence on the change in crack width in the structure, whereas variations in temperature could significantly influence the crack width with an average Pearson correlation of around 0.8. In addition, the crack width generally decreases with an increase in rainfall. Furthermore, a relatively low frequency (<25 Hz) induced by the traffic could result in a relatively larger crack width.

## 1. Introduction

The structural health of underground road structures in reclamation areas is affected by the vibrations induced by vehicles and the deterioration rate of construction materials, which is influenced by a range of environmental factors such as tide levels, rainfall and temperature throughout the year [1,2]. For road authorities, continuous maintenance and inspection of underground road structures are crucial and should be performed periodically to enhance the durability of these structures, which are an important component of transport infrastructure sustainability. 

The structural health monitoring (SHM) of underground road structures requires the development of advanced monitoring techniques and data processing methodologies. Hong et al. [3] investigated the effectiveness and efficiency of fiber Bragg grating (FBG) sensors in the monitoring of underground structures. A recent tunnel monitoring work using electronic theodolite successfully detected the movement of a large tunnel section along the tunnel axis, which resulted in delayed deformation of other previously stabilized sections and tunnel necking regularity [4]. Using vibrating wire sensors, Sircoulomb et al. [5] monitored crack development in an underground corridor structure by developing a real-time warning system for crack formation in underground structures. To further understand the deformation mechanism of underground tunnels, Walton et al. [6] obtained detailed deformation information on the surface of a tunnel using a ground laser scanner. In addition, previous experimental studies revealed that FBG sensors have stronger anti-interference abilities and higher monitoring accuracies than traditional sensors [7,8,9].

Data processing plays an important role in the structural health of underground road structures. The Braided Cooperative Reliable Transport (BCRT) is a data processing algorithm which is widely used to analyze the collected data with high efficiency [10]. To overcome the difficulties in analyzing the large amount of collected data, Zhong et al. [11] proposed an improved parallel clustering algorithm based on K-means. In addition, to quantify the nonlinear relationship between intrinsic soil parameters and tunnel convergence, the monitoring data can be analyzed using a methodology based on the support vector machine (SVM) and a Back-Propagation (BP) neural network algorithm [12,13]. Further, a K-means clustering method has been developed to determine the optimal number of clusters by investigating the relationship between mixed ratio, tunneling parameters and silhouette evaluation [14]. To identify the critical factors that affect the structural condition of underground structures, Bossi et al. [15] analyzed the influence of landslides on crack development in tunnels, while Qin et al. [16] studied the effects of continuous rainfall on the surrounding rock deformation in shallow-buried highway tunnels. In addition, to investigate the influence of seismic loading on the tunnels, a numerical model was proposed to predict the thrust generated during the slip process of the tunnel lining by measuring the slip between the tunnel lining and the ground soil [17]. However, to date, traditional monitoring techniques, such as resistance, inductive and potentiometric sensors, have poor anti-jamming ability and lower sampling frequency for long-term monitoring of underground structures in a complex environment. With a sampling frequency of 1000 Hz, fiber Bragg grating sensing monitoring technology has the capability to monitor multiple critical factors that affect the structural conditions of underground structures simultaneously through continuous long-term data collection.

In this study, an integrated remote real-time SHM system, including a remote real-time monitoring system and a developed data processing model, was developed to conduct condition assessment of composite road box-type structures in reclamation areas using fiber Bragg grating sensing monitoring technology by encapsulating the FBG sensors with stainless steel and adhesives. Taking a composite road box-type structure project in a reclamation area in Southern China as a case study, the developed system was implemented to investigate the effects of the change in tides, rainfalls, temperature and vehicle induced vibrations on the degradation of the structure simultaneously.

## 2. Experimental Study

**Figure 1:**
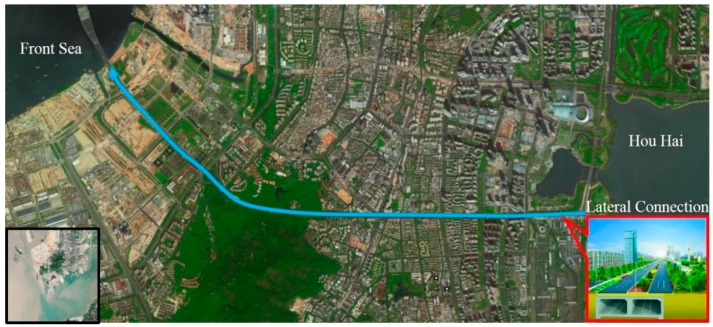
Population distribution and cross-sectional view of the composite road box-type structure.

In the present study, a 4.48 km long passage project in Southern China was investigated. The project consists of a 3.08 km long underground composite box-type structure which is located in a reclamation area with a six-lane highway on top of the structure. As shown in Figure 1, the underground composite box-type structure is comprised of three 6 m high compartments (13.2 m wide for the left and right compartments and 1.2 m wide for the middle one). The roof of these three compartments is around 1.0 m thick, and the thicknesses of the middle and side wall are 0.4 and 0.85 m, respectively. As can be seen from Figure 1, the structural condition of the composite road box-type structure is critically important for the transport system in the municipal district. The geomorphic units in the reclamation area of the underground road box-type structure are mainly sea terraces and sea floodplains with a thick untreated silt clay layer (4–10% organic matter) in the north, which is under-consolidated and highly compressible. These soil conditions make the composite road box-type structure prone to uneven settlement resulting from tides, rainfall, temperature and vehicle vibrations, and therefore it becomes of critical importance to continuously assess the condition of the structure.

### 2.1. Structural Health Monitoring of the Underground Box-Type Structure Using FBG Sensors

**Figure 2:**
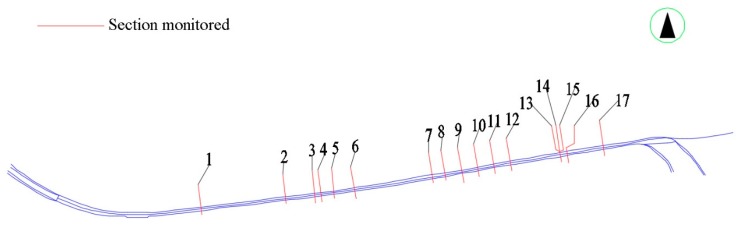
Schematic diagram of the 17 sections monitored in this study.

As shown in Figure 2, the deformation, crack development and ambient temperature of 17 sections of the box-type structure were monitored using FBG sensors. The selection criteria for these 17 sections were based on (1) maximum possible uneven settlements predicted by the numerical simulations, (2) the frequency of occurrence of cracks in the pavement of the road above a section, (3) the occurrence of bubbles and seepage in the ceiling of a section and (4) a section that goes across the subway tunnel.

**Figure 3:**
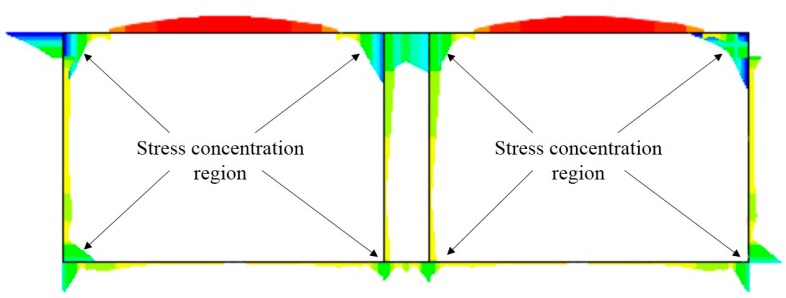
Stress nephogram of the cross-section of the tunnel under normal operation conditions.

**Figure 4:**
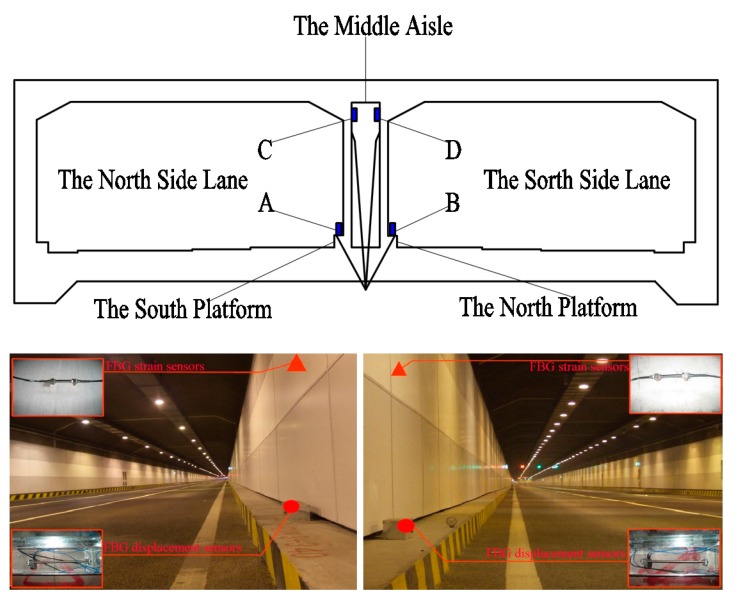
The positions of the fiber Bragg grating (FBG) sensors installed in underground box-type structure.

As shown in Figure 3, the numerical simulation results show that the stress concentration regions are mainly located in the top and bottom corners of the tunnel. Thus, the FBG sensors were installed in these positions (Figure 4). In addition, it has been observed that most of the cracks normally occur near the middle of the two lanes and drainage ditches of the highway above the box-type structure, so the installation of FBG displacement sensors was mainly concentrated near the middle passage, i.e., displacement sensors at A and B, respectively, and strain sensors at C and D, respectively. Further, the FBG temperature sensors were installed in each of the sections to monitor the temperature inside the box-type structure.

**Figure 5:**
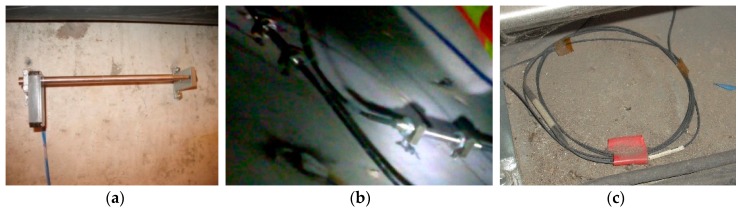
The details of FBG sensors: (**a**) Displacement sensor, (**b**) strain sensor and (**c**) temperature sensor.

The details of the different FBG sensors are shown in Figure 5. For FBG displacement sensors the measurement range was 20 mm, the resolution was less than 0.1% FS (full scale), the accuracy was less than 0.3 FS and the central wavelength of the grating was between 1525 and 1565 nm (10 nm = 10^−8^ m). For FBG strain sensors the test resolution was less than 0.1% FS, the precision was less than 2% FS, the working temperature range was between −20 and +50 °C, the strain measurement range was between −1000 and +1000 µε and the center wavelength shift range was between 1.5 and 1.8 nm. As FBG sensors were installed on the inner surfaces of the box-type structure, the effects of tides and rainfall could not be captured. Further, for FBG temperature sensors the range of measurement was between −50 and +80 °C, the temperature coefficient was 0.1 °C/pm (1 pm = 10^–12^ m), the measuring accuracy was less than 1 °C and the central wavelength of the grating was between 1525 and 1565 nm. The types and quantities of fiber grating sensors are summarized in Table 1. The integrated monitoring system consists of FBG and temperature sensors connected in series, so that the change of wavelength in the FBG strain sensors can be compensated for through consideration of the temperature field by measuring the wavelength change in the temperature sensors.

### 2.2. Remote Real-Time Monitoring System

With the development of modern communication technology and the wide application of computer technology, unattended remote monitoring systems have become a research focus in academia and engineering fields in the world [18]. The system enables the analysis of the structural conditions of the structures, remotely, based on the collected data.

#### 2.2.1. Network Architecture

**Figure 6:**
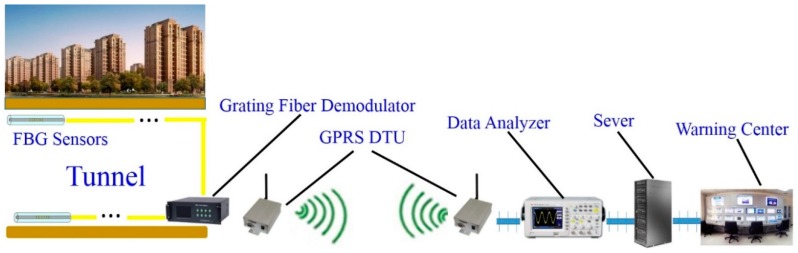
The details of the platform for remote data collection and processing.

**Figure 7:**
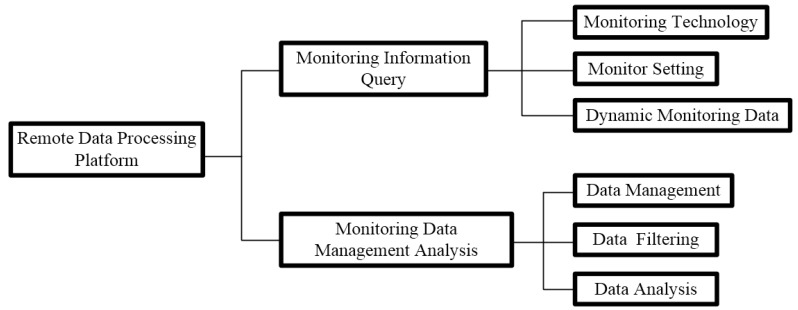
Network structure design of a remote data processing platform.

As the underground tunnel project is located in a reclamation area, a remote data processing platform was established in this study for data collection using an FBG demodulator (SM130-700) which transmits signals by combining a GPRS (General Packet Radio Service) DTU (data terminal unit) with an industrial PC (IPC-810-a), as shown in Figure 6. As shown in Figure 7, the platform consisted of two main modules (i.e., information query and data management).

#### 2.2.2. Data Transmission

**Figure 8:**
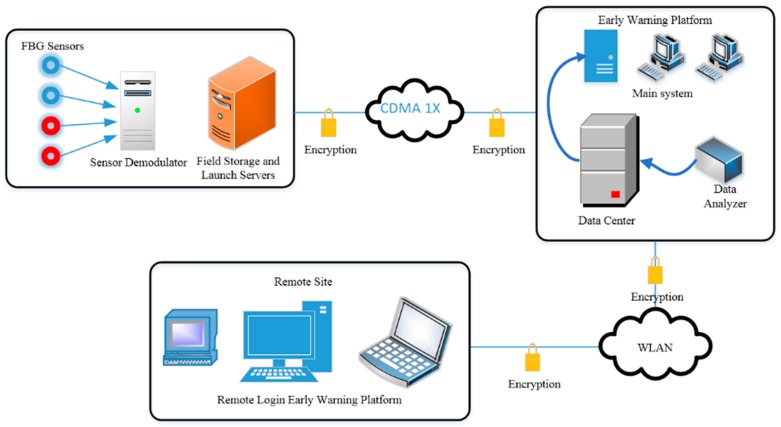
The structure of remote transmission network.

Because a large amount of data was collected by sensors in the field and had to be transmitted back to the data monitoring center, which is located more than 120 km from the field, a wireless sensor network was established in this study by adopting GPRS/CDMA (code-division multiple access) 1x mobile data communication technology in combination with a DTU and DVB (digital video broadcasting) antenna. The remote wireless transmission and control of monitoring data were realized with a lapping wireless router, DVB antenna and industrial computer. In addition, different types of data conversion technology were developed. The structure of the remote transmission network is shown in Figure 8.

#### 2.2.3. Stability Analysis System

**Figure 9:**
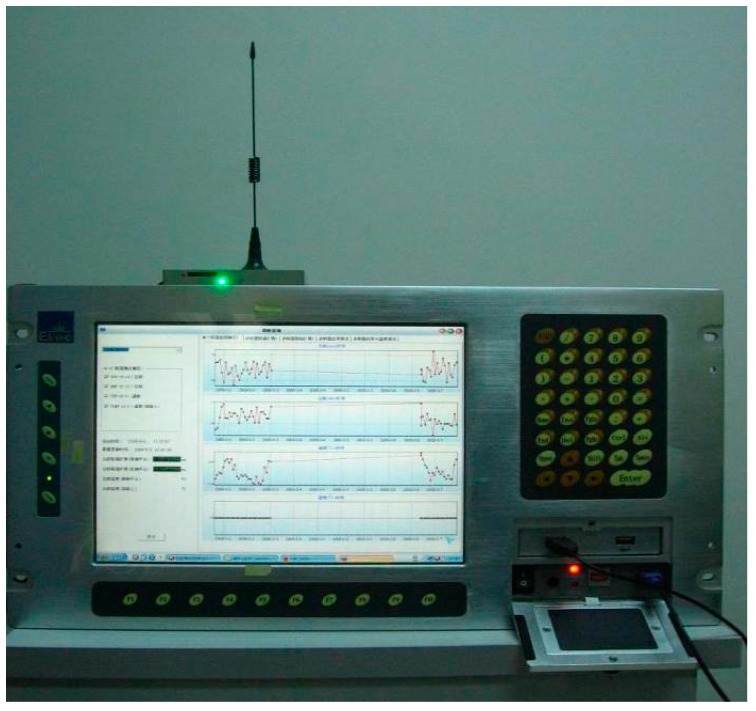
CWST01 stability analysis system for remote fiber optic sensors.

**Figure 10:**
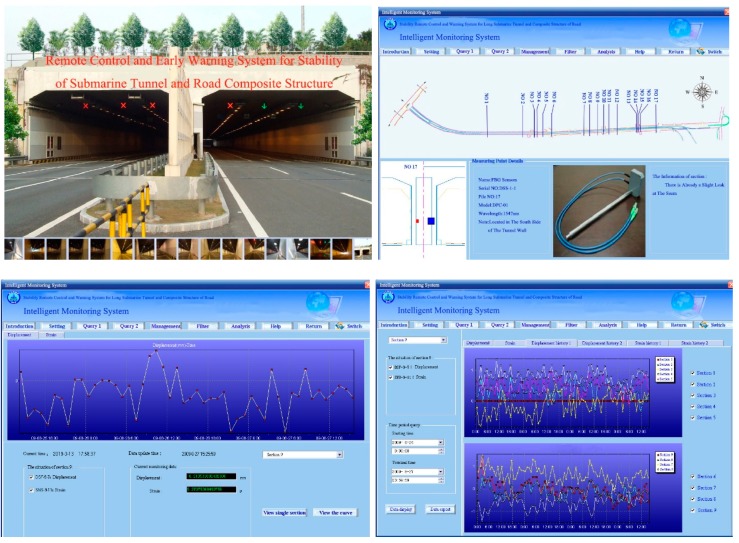
Health condition monitoring system for the compound road box-type structure in the reclamation area.

As shown in Figure 9, a CWST01 remote optical fiber sensing stability system was developed. The system consists of a GPRS/CDMA 1x mobile data communication module, wireless transceiver module and mass data fast data processing module. CWST01 realizes the remote transmission of monitoring data in a rapid and reliable manner. The overall remote data processing platform is shown in Figure 10.

### 2.3. The Development of a Time Scale Method

The purpose of this section was to develop a time scale method for investigating how critical factors (e.g., tides, rainfall and vehicle vibration) affect the structural conditions of the underground road box-type structure. The time scales and details of the critical factors investigated in this study are listed in Table 2.

To determine the time scales of different influencing factors, the time-dependent changes in temperature and crack width, as well as their correlations, were extracted by using the least squares method and Pearson correlation coefficient. In addition, the influences of tide and rainfall on the tunnel through changes in temperature were also investigated. Furthermore, the correlation between crack propagation and vehicle vibration was also studied using the higher order least squares method.

For a group of obtained data (*x_t_*, *y_t_*), the minimization of the sum of the squares of the differences *δ*(*a_i_*) between the function *F*(*x_t_*) and *y_t_* can be expressed in Equations (1) and (2) as:

(1) $$\delta (\alpha_{i}{)}_{\min}=\sum {\left(y_{t}-F(x_{t})\right)}^{2}$$

(2) $$\left[\begin{array}{l} m\;\;x_{i}\;\;\ldots \;\;x_{i}^{n}\\ x_{i}\;\;x_{i}^{2}\;\;\ldots \;\;x_{i}^{n+1}\\ \ldots \;\;\ldots \;\;\;\;\ldots \;\;\;\ldots \\ x_{i}^{n}\;\;x_{i}^{n+1}\;\;\ldots \;\;x_{i}^{2n} \end{array}\right]\left[\begin{array}{l} a_{0}\\ a_{1}\\ \ldots \\ a_{n} \end{array}\right]=\left[\begin{array}{l} y_{i}\\ x_{i}y_{i}\\ \;\ldots \\ x_{i}^{n}y_{i} \end{array}\right]$$

where *a_i_* (*i* = 1, 2, 3 … *n*) is the parameter and *m* is the group number of the data (*m* > *n*). By solving Equation (2), the optimal fitted Equation (3) can be obtained:

(3) $$f(x)=a_{0}+a_{1}x+a_{2}x^{2}+\ldots +a_{n}x^{n}$$

In order to explain the influence law for crack width variation theoretically influenced by time-dependent critical parameters (i.e., temperature, water head, precipitation and vehicle flow) on the corresponding time scale, this paper first constructs an equation (*S_overall_*) for the width variation of a crack in a section under the action of various factors. The second-order partial derivative was then calculated for different factors, and the degree of influence on crack width variation was judged by concave up or concave down. At the same time, the first-order partial derivative was used to solve for the critical point of influence on the curve of the change of crack width variation. The detailed process of derivation is as follows:

(4) $$S_{overall}=\int_{0}^{t}S_{T}dt+\int_{0}^{t}S_{H}dt+\int_{0}^{t}S_{I,R}dt+\int_{0}^{t}S_{V}dt$$

where *T* is the temperature, *S_T_* is the fitted equation for the effect of temperature on the width variation of a crack, *H* is the water head, *S_H_* is the fitted equation for the effect of tide on the width variation of a crack, *I* is the rainfall intensity, *R* is the precipitation, *S_I,R_* is the fitted equation for the effect of rainfall on the width variation of a crack, *V* is the vehicle flow and *S_V_* is the fitted equation for the effect of vehicle vibration on the width variation of a crack.

Under different time scales, the conditions satisfied by the definition of critical time-dependent crack width in the box-type structure as follows.

- Effects of vehicle vibration on crack width in the box-type structure (*t_car_*, time scale: Seconds)
- Critical time-dependent crack width can be defined when $\frac{\partial S_{overall}}{\partial t_{car}}=0$($\frac{\partial^{2}S_{overall}}{\partial t_{car}^{2}}<0$);
- Effects of temperature on crack width in the box-type structure (*t_temp_*, time scale: Hours)
- Critical time-dependent crack width can be defined when $\frac{\partial S_{overall}}{\partial t_{temp}}=0$($\frac{\partial^{2}S_{overall}}{\partial t_{temp}^{2}}<0$);
- Effects of tide on crack width in the box-type structure (*t_tide_*, time scale: Months)
- Critical time-dependent crack width can be defined when $\frac{\partial S_{overall}}{\partial t_{tide}}=0$($\frac{\partial^{2}S_{overall}}{\partial t_{tide}^{2}}<0$);
- Effects of rainfall on crack width in the box-type structure (*t_rainfall_*, time scale: Days)
- Critical time-dependent crack width can be defined when $\frac{\partial S_{overall}}{\partial t_{rainfall}}=0$($\frac{\partial^{2}S_{overall}}{\partial t_{rainfall}^{2}}<0$).

The width variation of cracks influenced by vehicle vibration, which is non-stationary in nature, were processed using short-time Fourier transform (STFT) analysis. STFT is a time–frequency localization method which can be defined as:

(5) $$F(t,f)=\int_{-\infty }^{\infty }x(u)g^{*}(u-t)e^{-j2\pi fu}du$$

where *g*(*t*) is the window function and *x*(*t*) is the vehicle vibration signal.

By discretizing the STFT and sampling it at points (*m*Δ*t*, *n*/(*N*Δ*t*)) in the time–frequency domain, we obtain:

(6) $$F(m,n)=\sum_{k=0}^{N-1}x(k\Delta t)g^{*}(k\Delta t-m\Delta t)e^{-j2nk\pi /N}$$

where *x*(*k*) is the discrete form of the signal, Δ*t* is the sampling interval and *N* represents the sampling points, *m*, *n* = 0, … *N* − 1. A window function (*g*(*t*)) could exist to analyze the width variation of cracks influenced by vehicle vibration in the time and frequency domains simultaneously, and ultimately the effects of vehicle vibration on the tunnel.

To quantify the correlation between temperature change and crack propagation, the Pearson correlation coefficient (*ρ_X,Y_*) [19] was adopted:

(7) $$\rho_{X,Y}=\frac{\sum \left(X_{i}-\bar{X})(Y_{i}-\bar{Y}\right)}{\sqrt{\sum {\left(X_{i}-\bar{X}\right)}^{2}{\left(Y_{i}-\bar{Y}\right)}^{2}}}$$

The value of *ρ_X,Y_* is between −1 and +1. The larger |*ρ_X,Y_*| is, the stronger the correlation between vectors *X* and *Y* becomes. Thus, the correlation between temperature change and width variation of cracks can be established.

## 3. Results and Discussion

The data collection mainly focused on the period from September to October, which is the rainy season in South China and has complex weather patterns. Thus, the structural performance of the underground structures needs to be closely monitored during this period. The collected monitoring data from 17 sections was systematically analyzed, and the effects of vehicle induced vibration, temperature, tide and rainfall on time-dependent changes to crack width were investigated. The results show that the time-dependent changes to crack width were affected by the vehicle induced vibration on the time scale of seconds, temperature through the nature of thermal expansion of concrete on the time scale of hours, the tide on the time scale of days and rainfall on the time scale of months. In addition, it was found that rainfall could indirectly affect the influence of temperature on crack width through changes in the groundwater level.

### 3.1. Influence of Variation in Tide Height and Temperature on the Change of Crack Width in the Box-Type Structure

**Figure 11:**
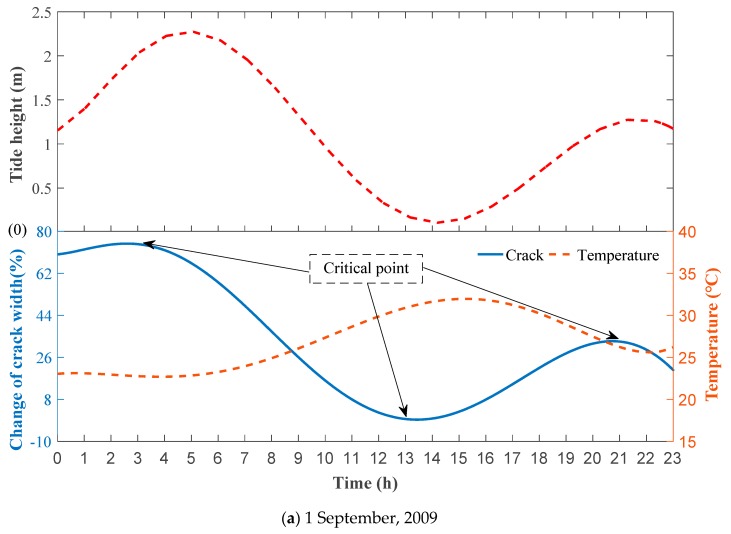

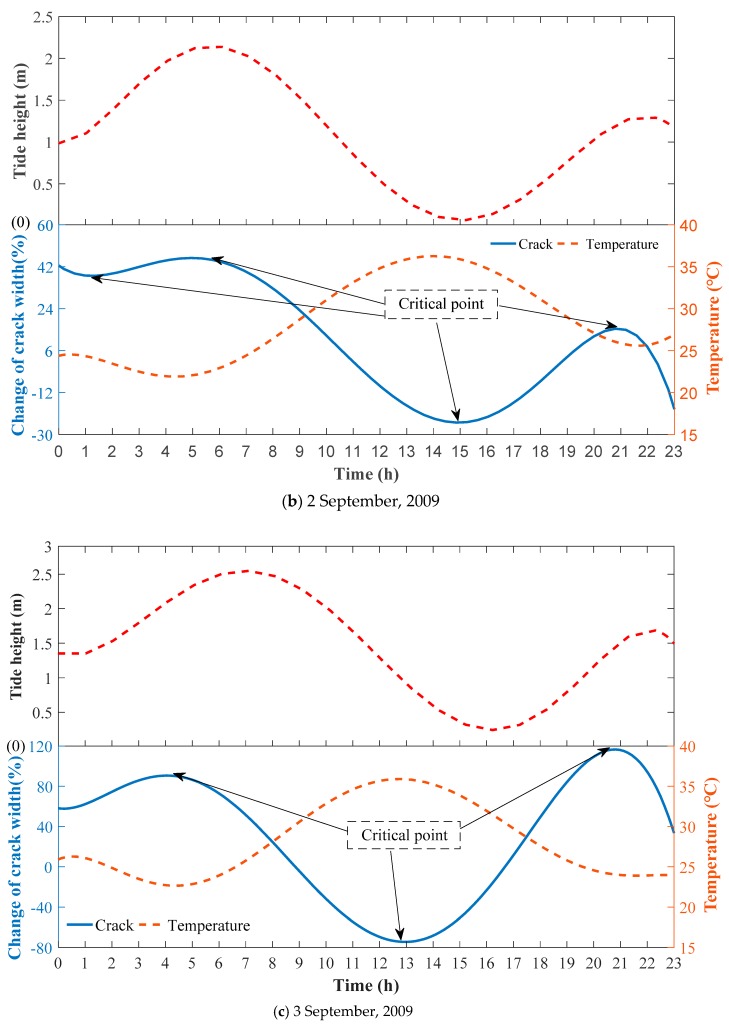
Influence of variation in tide height and temperature on the change in crack width in the box-type structure (Section 1) on (**a**) 1, (**b**) 2 and (**c**) 3 September, 2009.

As the time-dependent changes in crack width in Section 1, Section 3 and Section 4 due to the changes in temperature are similar, Section 1 was selected as a case study. Figure 11 shows the time-dependent variations in crack width, temperature and tides on September 1, 2 and 3 in 2009. In this study we investigated the effects of vehicle induced vibration, temperature, tide and rainfall on the time-dependent change in the width of an existing crack. A negative value of the change (%) represents a decrease in crack width, while a positive value of the change (%) represents an increase in crack width.

During the tidal variation, the change of temperature had a great effect on the change of time-dependent crack width (Pearson correlation coefficient >0.6). For example, the variation of temperature can significantly affect the change in crack width (e.g., 120% increase in crack width) with Pearson correlation coefficients of 0.65, 0.84 and 0.86 for September 1, 2 and 3 in 2009, respectively. However, our results show that there is no strong correlation between the tidal variation and change in crack width. The change in crack width due to the change in temperature is a relatively slow process. Thus, the Pearson correlation analysis between temperature change and crack propagation were performed between the first and the third of September.

**Figure 12:**
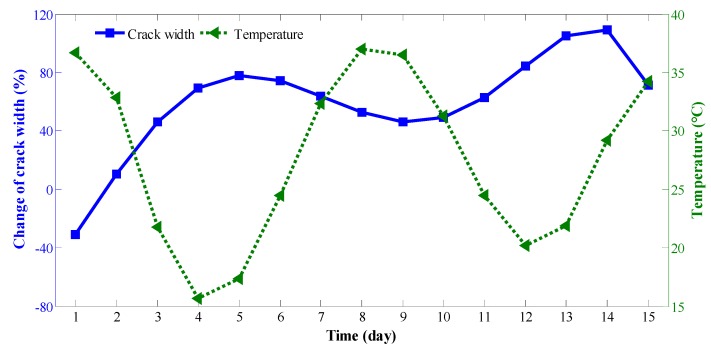
Comparison of width variation of cracks in Section 1 and temperature data from 1 September to 15 September, 2009.

To study the influence of temperature on the continuity of cracks, 15 days of uninterrupted monitoring were carried out on the cracks on the southern side of Section 1, as shown in Figure 12. By comparing the monitoring data for crack width with the temperature in the graph, the curve of width variation of the crack shows strong correlation with the temperature change curve. When the temperature increases, the width variation of cracks decreases, whereas when the temperature decreases, the width variation of cracks increases. Additionally, the temperature change effect on the width variation of cracks at high levels of the box-type structure is more obvious than that at low levels. Therefore, temperature has a greater impact on crack width, and is the main factor affecting crack changes. Simultaneously, the changes in cracks and temperature are not simultaneous, and there is a certain delay.

### 3.2. Influence of Variation of Rainfall on the Change in Crack Width in the Box-Type Structure

**Figure 13:**
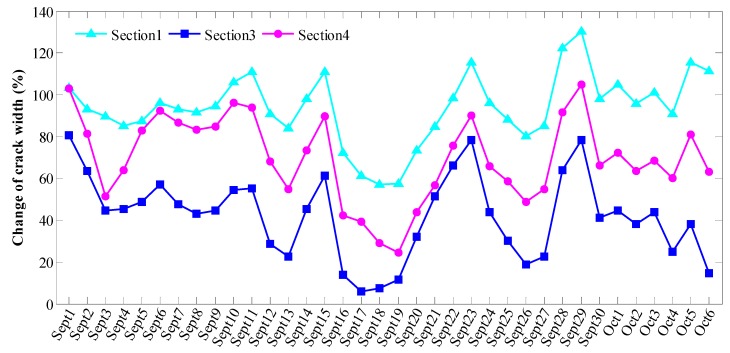
Time-dependent change in crack width in Section 1, Section 3 and Section 4 during the period from September to early October, 2009.

**Figure 14:**
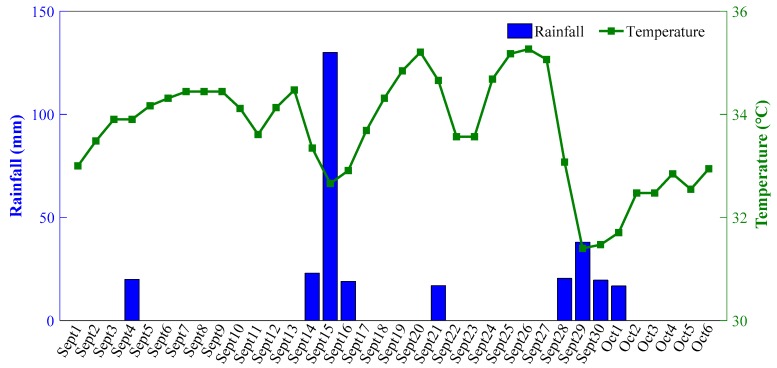
Time-dependent rainfall and temperature during the period from September to early October, 2009.

**Figure 15:**
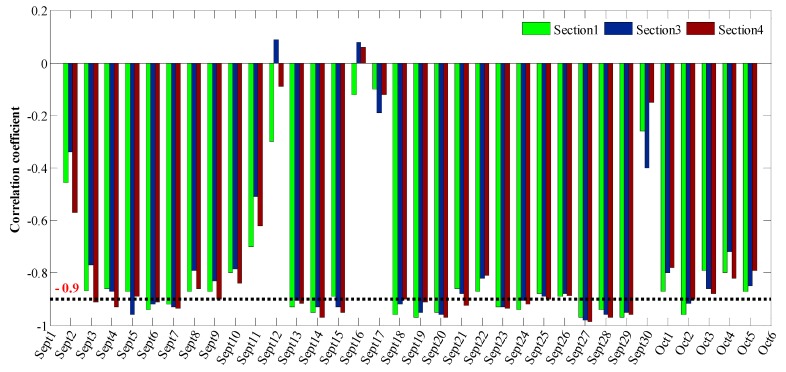
Time-dependent correlation coefficient describing the relationship between the change in crack width (Section 1, Section 3 and Section 4) and temperature during the period from September to early October, 2009.

Rainfall may affect soil moisture, which may in turn affect the temperature of underground structures, and ultimately the crack width. Figure 13 shows the time-dependent crack width in Section 1, Section 3 and Section 4 during the period from September to early October in 2009. It shows that the crack width varied from 0.3 to 1.8 mm and the frequent retractable movement of cracks will aggravate the structural fatigue and impact the stability. In addition, the time-dependent rainfall and temperature during the period from September to early October in 2009 are shown in Figure 14. This demonstrates that a high rainfall could result in a decrease in temperature. Figure 15 shows the time-dependent correlation coefficient describing the relationship between the changes in crack width (Section 1, Section 3 and Section 4) and temperature during the period from September to early October in 2009. These results indicate that there is a strong negative relationship between rainfall and the variation in crack width.

### 3.3. Influence of Vehicle Induced Vibration on the Change in Crack Width in the Box-Type Structure

**Figure 16:**
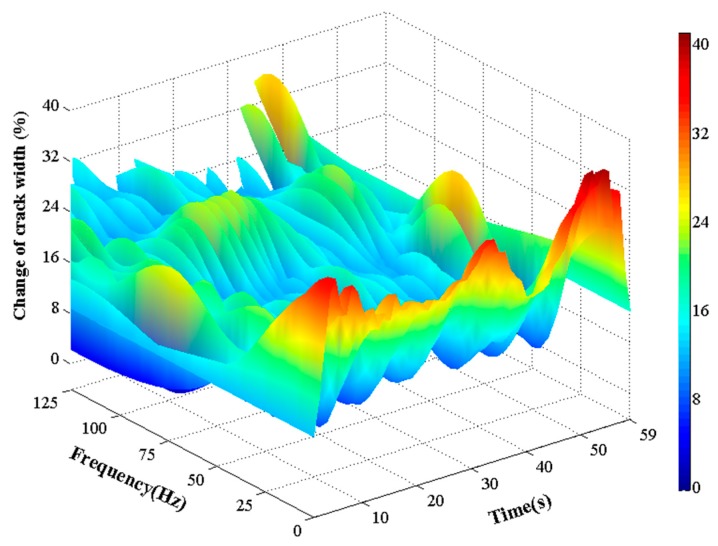
Influence of vehicle induced vibration on the time-dependent change in crack width in Section 3 on 28 September, 2009.

So far, there are limited studies on investigating the effects of vehicle vibration on tunnel structures. Figure 16 shows the influence of vehicle induced vibration on the time-dependent change in crack width in the box-type structure. One-minute monitoring data was collected due to the relatively short passing time of vehicles. The results from the time–frequency analysis show that a relatively low frequency of vibration (i.e., less than 25 Hz) will result in a larger increase in crack width. Figure 16 also shows that when vehicles are close to the box-structure, the width of a crack will increase. Conversely, the crack width gradually returns to its original level.

## 4. Conclusions

In the present study, we quantitatively assessed the structural condition of a composite road box-type structure in a reclamation area to investigate the influence of environmental factors (e.g., tide levels, rainfalls and temperature) and vehicle induced structural vibration. The following are the major conclusions:

- The change of tide levels has little influence on the change in crack width.
- Variations in temperature can significantly influence the crack width (e.g., 120% increase in crack width) with Pearson correlation coefficients of 0.65, 0.84 and 0.86 for August 1, 2 and 3 in 2009, respectively.
- There is a strong negative correlation between rainfall and the change in crack width.
- A relatively low frequency of vibration (i.e., less than 25 Hz) can result in a relatively large increase in crack width.

## Figures and Tables

**Table 1:** Types and quantities of fiber Bragg grating sensors used in this study.

Types of FBG Sensors	Quantities of Sensors
strain sensor	24
displacement sensor	17
temperature sensor	3
total	44

**Table 2:** Time scales and details of the critical factors investigated in this study.

Parameters	Time Scale	Details
tide	day	Groundwater level rise and fall
rainfall	month	Groundwater level rise and fall
vehicle vibration	second	Amplitude changes
temperature	hour	Thermal expansion
